# Mechanisms governing bystander activation of T cells

**DOI:** 10.3389/fimmu.2024.1465889

**Published:** 2024-11-27

**Authors:** Mohammed Yosri, Mohamed Dokhan, Elizabeth Aboagye, Mouhamad Al Moussawy, Hossam A. Abdelsamed

**Affiliations:** ^1^ The Regional Center for Mycology and Biotechnology, Al-Azhar University, Cairo, Egypt; ^2^ Immunology Center of Georgia (IMMCG), Medical College of Georgia (MCG), Augusta University, Augusta, GA, United States; ^3^ Starzl Transplantation Institute, School of Medicine, University of Pittsburgh, Pittsburgh, PA, United States; ^4^ Department of Physiology, Augusta University, Augusta, GA, United States

**Keywords:** T cells, bystander, activation, cytokines, peptide/MHC complex, cross-reactivity

## Abstract

The immune system is endowed with the capacity to distinguish between self and non-self, so-called immune tolerance or “consciousness of the immune system.” This type of awareness is designed to achieve host protection by eliminating cells expressing a wide range of non-self antigens including microbial-derived peptides. Such a successful immune response is associated with the secretion of a whole spectrum of soluble mediators, e.g., cytokines and chemokines, which not only contribute to the clearance of infected host cells but also activate T cells that are not specific to the original cognate antigen. This kind of non-specific T-cell activation is called “bystander activation.” Although it is well-established that this phenomenon is cytokine-dependent, there is evidence in the literature showing the involvement of peptide/MHC recognition depending on the type of T-cell subset (naive vs. memory). Here, we will summarize our current understanding of the mechanism(s) of bystander T-cell activation as well as its biological significance in a wide range of diseases including microbial infections, cancer, auto- and alloimmunity, and chronic inflammatory diseases such as atherosclerosis.

## 1 Introduction

The Oxford Language Dictionary defines “bystander” as a person who is physically present during an ongoing specific event but not directly involved in it. Nature is full of bystander examples that might be of symbiotic benefits or deleterious consequences. For instance, during photosynthesis, green-leaved plants release oxygen into the atmosphere as a by-product of the process (1). Consequently, eukaryotes can perform cellular respiration using released oxygen to produce their cellular energy. This interconnected chain of events represents a clear example of a bystander effect where eukaryotic cells, not necessarily participating directly in photosynthesis, get affected beneficially from using their oxygen by-product.

**Figure 1:**
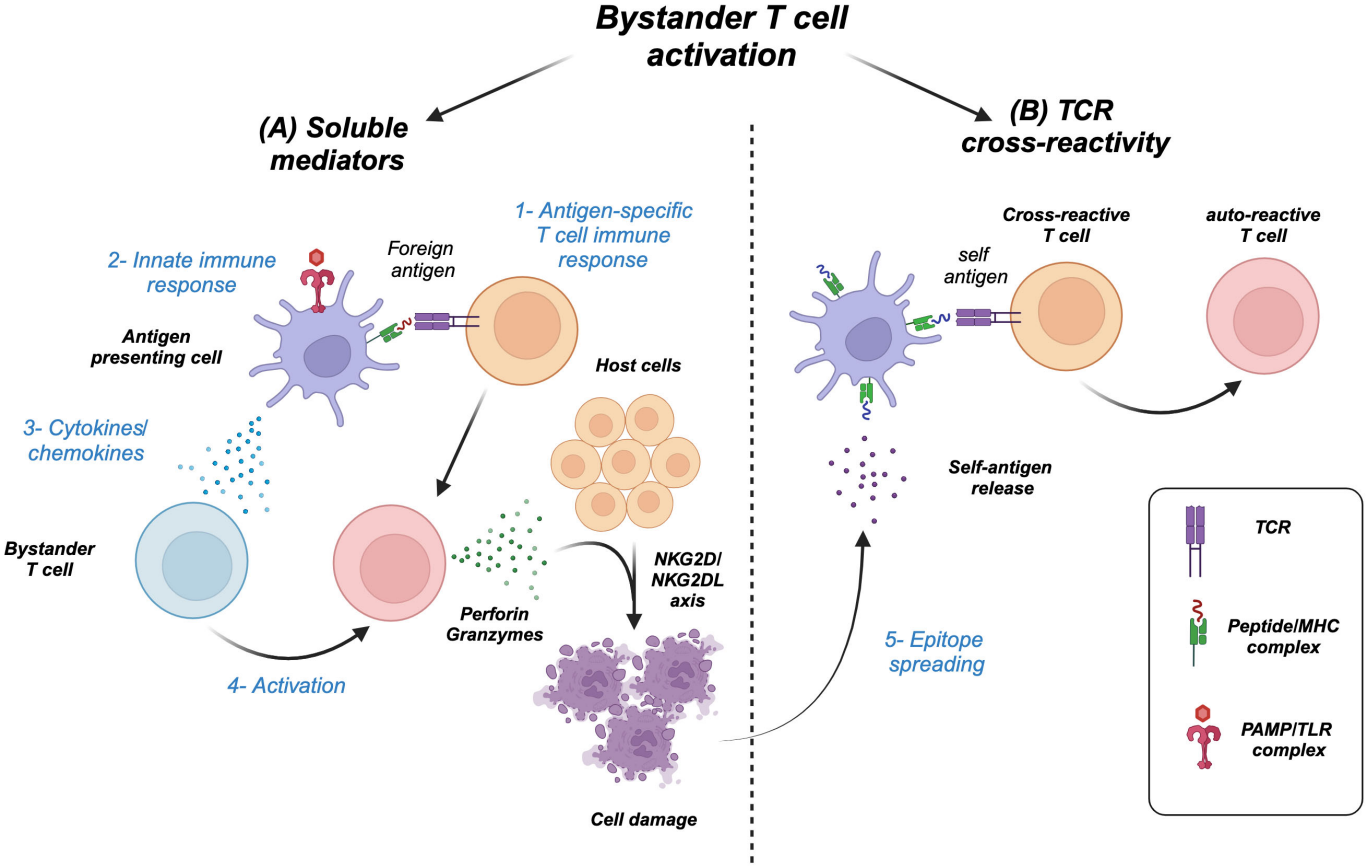
Types of T cell bystander activation. **(A)** 1) Antigen-specific immune response and/or 2) innate stimulation of antigen-presenting cells results in 3) a wave of cytokines/chemokines that 4) activate bystander T cells, which can cause host cell damage through effector molecules (granzymes/perforin) and NK receptor–ligand interaction (NKG2D/NKG2DL). **(B)** 5) The damage can release new self-antigens, a process called epitope spreading, which in turn activates cross-reactive T cells including initial antigen-specific T cells to become autoreactive.

Similarly, cytokines as by-products of functional immune responses (2, 3) can activate a wide range of immune cells including T cells but not necessarily those specific to the initial non-self antigen. In other words, antigen non-specific T cells that happen to be present during an ongoing immune response can be activated by cytokines as bystanders, a phenomenon known as “bystander T cell activation” (**Figure 1A**) (4–6). However, the high cost of this process can lead to “epitope spreading,” where collateral damage of the host cells results in the release of new antigens and the activation of T cells within the microenvironment leading to autoimmunity (**Figure 1B**) (7–9).

Although it is widely accepted that bystander memory T cell activation is cytokine-dependent (10–12), there is evidence supporting the role of the self-peptide/MHC complex along with cytokines in the activation of naive T cells (5, 13–17). In this review, we will shed light on the mechanisms of bystander T-cell activation (**Figure 1**). Furthermore, we will discuss the biological relevance of this process in autoimmunity, cancer, transplantation, microbial infection, and atherosclerosis.

## 2 Mechanisms of bystander T cell activation

The communication between different cell types in the body is mainly governed by at least two processes: 1) receptor–ligand interaction and (2) soluble mediators (18). Likewise, T cells can be activated through their T-cell receptor (TCR) via interaction with the peptide/MHC complex, presented by APCs (receptor–ligand complex) (19, 20) and/or soluble mediators including cytokines and microbial products (21–34), where both types of communication can contribute to the bystander activation of T cells.

Bystander T-cell activation is defined as the activation of T cells that are non-specific to the original antigen (35). In other words, during viral infections, there could be activation of T cells not necessarily specific to viral antigens (35). For instance, several studies demonstrated the activation of antigen non-specific T cells during mouse lymphocytic choriomeningitis virus (LCMV) infection (35–37). Additionally, LCMV-specific cytotoxic T cells (CTLs) undergo activation and proliferation following infection with unrelated viruses including murine cytomegalovirus (MCMV) and vaccinia virus (VV) (38).

### 2.1 Bystander T cell activation via soluble mediators versus cross-reactivity

Bystander T-cell activation can actually happen through two mechanisms: first, via activation of cross-reactive TCRs (**Figure 1B**) (39–42), and second, via activation through soluble mediators including cytokines (21–25, 27, 28, 30–33, 43) because of the strong immune response to the original foreign antigen (**Figure 1A**). Although the phenomenon of bystander T-cell activation is widely associated with stimulation via soluble mediators including cytokines, several follow-up studies demonstrated the involvement of peptide–MHC/TCR interaction in the process (15, 16, 44, 45). These studies served as an impetus for us to further understand the mechanisms regulating this process as well as the differential requirement for each mechanism that can be dependent on the T-cell subtype (naive vs. memory). Furthermore, we would like to clarify the concept of cross-reactivity and its relation to bystander T-cell activation. Cross-reactivity is simply the ability of a single TCR to recognize multiple peptide–MHC complexes, which was first hinted as a hypothesis by Matzinger and Bevan (46). Indeed, it is plausible for the immune system to develop such a feature of TCR degeneracy or so-called heterologous immunity to recognize a vast number of microbial epitopes, yet molecular mimicry could be the high cost of such capacity where an overlap between microbial and self-epitopes results in an immune response against self and the development of autoimmune diseases. A classic example of such a phenomenon is rheumatic carditis, where cardiac myosin is cross-reactive with a virulence factor related to group A streptococcus (GAS), *Streptococcus pyogenes*, called the M protein (47–49). One can speculate that the host-immune response against the bacteria damages the heart tissue by exposing new epitopes including myosin. Consequently, the initial T-cell immune response becomes cross-reactive against the self-proteins causing autoimmunity (**Figure 1B**). Additionally, we will discuss in the next sections, many types of autoimmune diseases that are characterized by the presence of bystander-activated antigen non-specific T cells in the inflamed/damaged organ, hinting to their activation either via the proinflammatory microenvironment and/or cross-reactivity. Yet, it is challenging to dissect bystander T-cell activation via cytokines versus cross-reactivity, as TCRs will have some degree of responsiveness to self, i.e., tonic signaling. In the following sections, we will discuss the mechanisms regulating bystander T-cell activation and their effect on naive and memory CD8 T cells.

#### 2.1.1 The role of microbial products and cytokines in naive and memory T-cell activation

The secretion of cytokines in a given microenvironment and the role of pathogen-associated microbial patterns (PAMPs) are two intermingled processes, where activation via PAMPs results in the secretion of cytokines. Consequently, pathogen recognition receptors (PRRs) are upregulated. PAMPs are known to bind directly to T cells through PRRs including Toll-like receptors (TLRs), acting as costimulatory signal, activating them to upregulate activation markers and secretion of cytokines (50–52). Indeed, TLR2 engagement in T cells enhances their effector functions and survival (26, 29), while TLR7 activation in T cells contributes to the pathogenesis of multiple sclerosis in a preclinical animal model (34). *In vitro* infection of splenocytes with *Burkholderia pseudomallei* bacteria results in rapid expression of IFNγ in CD8 CD44^hi^ (activated/memory) compared to naive CD8 CD44^lo^ cells (30). Furthermore, the Sprent lab showed that memory T cells were responsive to TLR ligands including poly I:C (TLR3 ligand) and LPS (TLR4 ligand), which can induce type I interferon and consequently can synthetize memory CD8 T cells to IL-15 and IL-2 by upregulation of IL-2/IL-15Rβ (CD122) (5, 53). To further examine the role of PAMPs on T-cell bystander activation *in vivo*, poly I:C was injected into naive mice mimicking viral infections, which resulted in the proliferation of polyclonal memory CD8 T cells in a TCR-independent manner (5).

**Figure 2:**
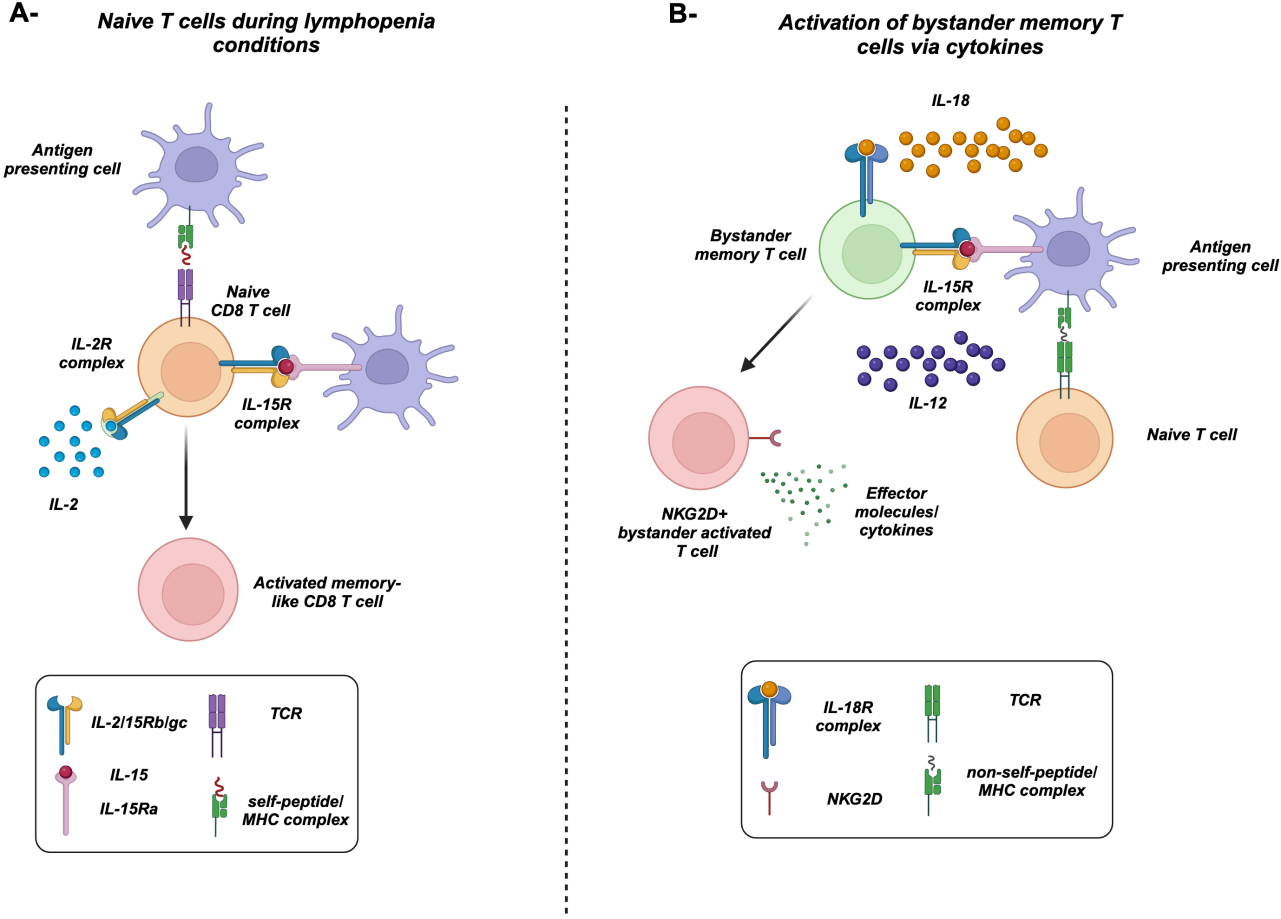
Bystander-activated memory T cells versus homeostatic proliferation of naive T cells. **(A)** Under lymphopenia conditions, naive CD8 T cells, recognizing a self-antigen presented by MHC-I, respond to the increased levels of IL-2 and cross-presented IL-15. They acquire a proliferative effector/memory-like phenotype. **(B)** Antigen-specific naive T cells recognize its specific antigen presented on MHC. In the meantime, IL-15 cross-presented by activated antigen-presenting cells, along with IL-12 and IL-18, activates bystander memory T cells in a TCR-independent manner. Activated memory cells upregulate the natural killer group 2D receptors (NKG2D) and produce high levels of effector molecules/cytokines including IFNγ along with granzymes and perforin.

**Figure 3:**
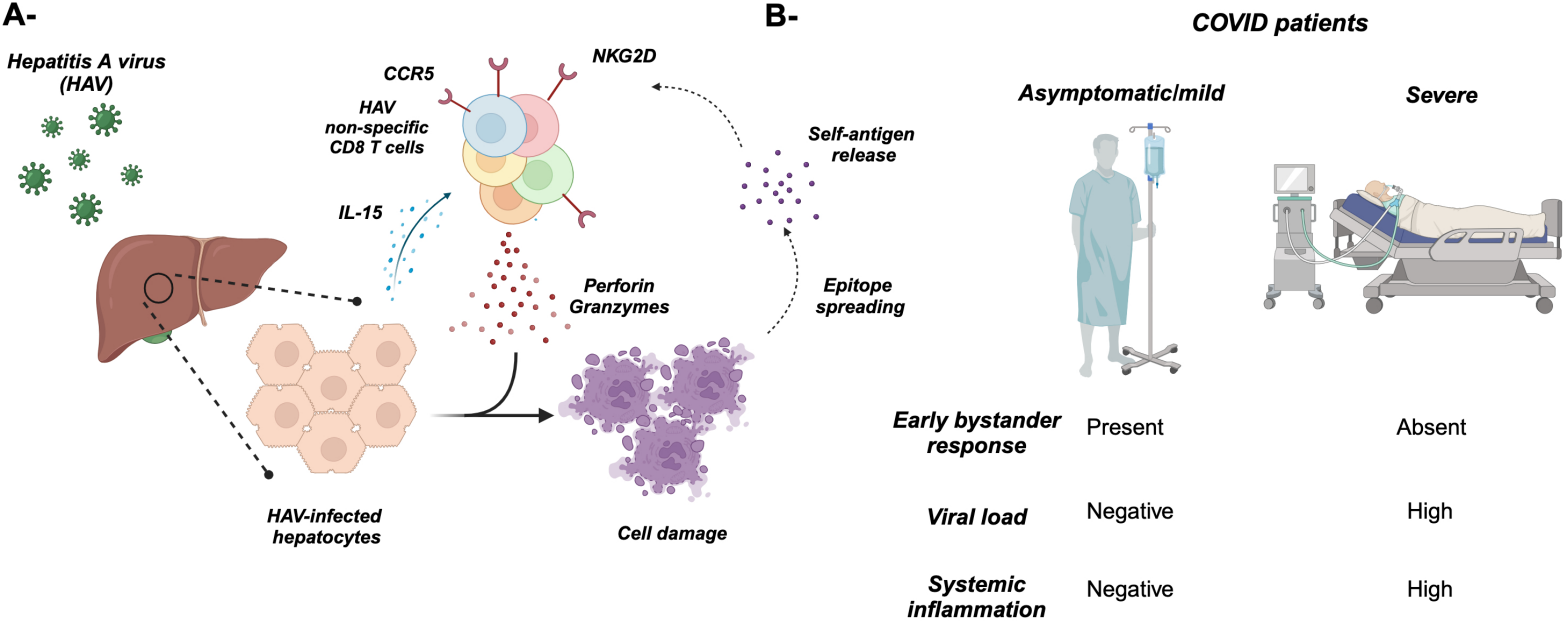
Bystander T cell activation during microbial infections. **(A)** Infection of hepatocytes with hepatitis A virus (HAV) results in the release of IL-15, which activates non-specific “bystander” CD8 T cells including EBV- and CMV-specific (depicted in different colors). These activated T cells cause damage to the hepatocytes through NKG2D and cytotoxic cytokines/molecules including perforin, granzymes, and IFNγ. The overall hepatocyte damage might result in the release of new self-antigens that activate T cells and cause autoimmunity through “epitope spreading” (dotted arrows). **(B)** Asymptomatic/mild COVD-19 patients are characterized by an early burst of bystander T-cell activation, which might protect them from disease progression to severe clinical presentation.

**Figure 4:**
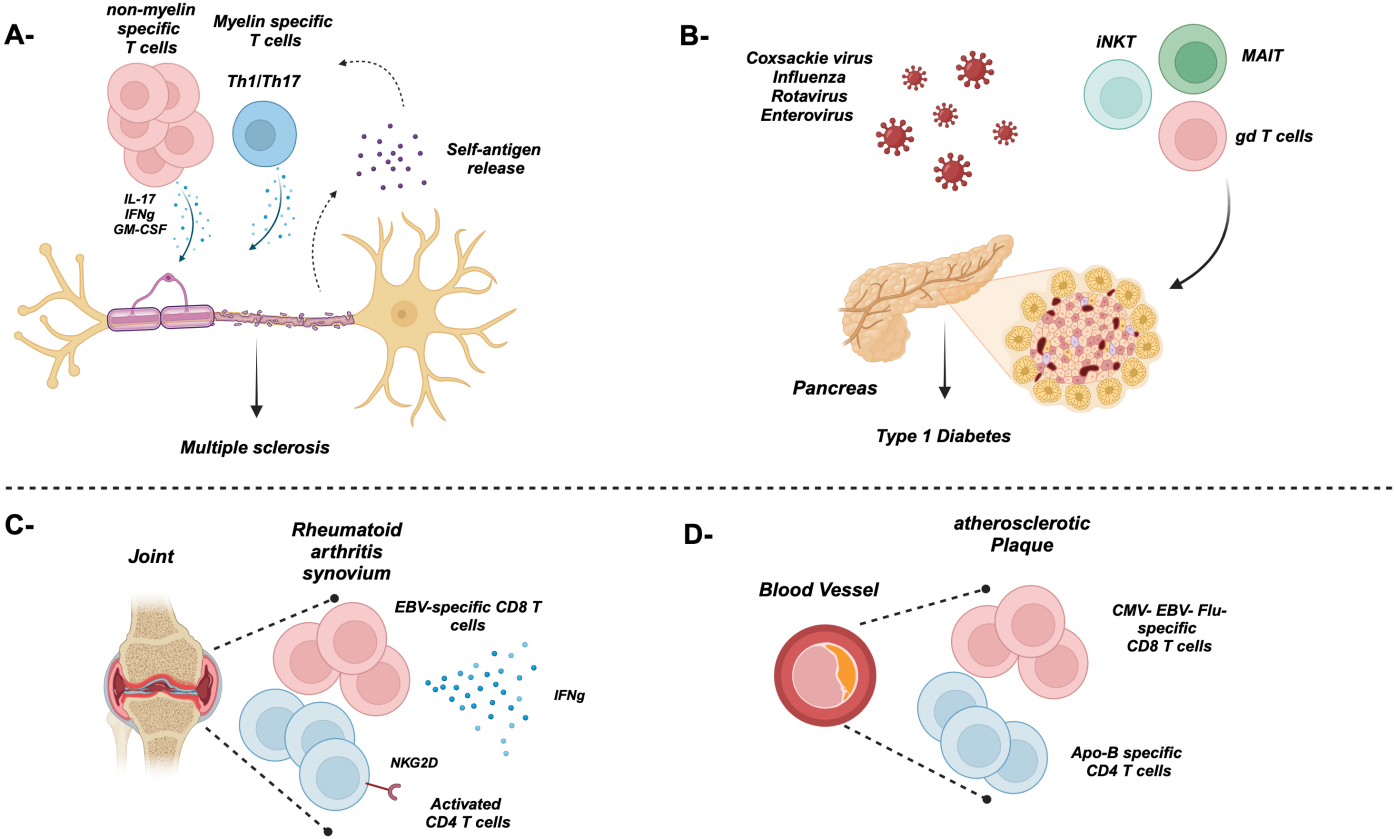
Bystander T cell activation during autoimmune diseases. **(A)** Myelin- and non-myelin-specific T cells contribute to the damage of the neuron’s myelin sheath during multiple sclerosis through proinflammatory cytokines. The release of new self-antigens might exacerbate the disease through the activation of T cells non-specific to the original antigen. **(B)** The Coxsackie virus as well as others can trigger and contribute to the damage of insulin-secreting beta cells in several ways. Furthermore, innate-like bystander T cells such as iNKT, MAIT, and gd T cells also play a pathogenic role in T1D. **(C)** Majority of T cells in the synovium are antigen non-specific including EBV- and CMV-specific, causing damage to the synovium through the secretion of proinflammatory cytokines. **(D)** An atherosclerotic plaque lining a blood vessel is rich in ApoB-specific and non-specific T cells.

Several studies examine the effect of the proinflammatory cytokines such as IL-12 and IL-18 as well as the common gamma chain cytokine IL-15 on bystander T-cell activation (**Figure 2B**) (21, 23–25, 28, 31, 43). For example, the combination of both IL-12 and IL-18 induced the proliferation and IFNγ expression from memory CD8 T cells (23, 28, 32). Furthermore, IL-12 augments the induction of IFNγ from T cells by enhancing the expression of IL-18R (54). Additionally, IL-15 alone in the absence of TCR stimulation can activate memory CD8 T cells to proliferate and acquire effector functions, while prolonged exposure enhances their cytotoxicity i.e., upregulation of perforin, granzymes, CD107a, granulysin, and NK receptors such as NKG2D (21, 24, 25, 31, 43). NKG2D can mediate direct cell killing in the absence of TCR stimulation (4, 55), recognizing its ligands including stress-induced proteins MICA and MICB in humans and H60 and Mult1 in mice (56). This binding results in the activation of downstream signaling through DAP10/12. The killing of target cells can result in the release of self-antigens that can activate bystander T cells amplifying an autoimmune response through epitope spreading (**Figures 1A**, **3A**, **4A**). For instance, in celiac disease, an autoimmune disease, intestinal epithelial cells express IL-15, which upregulates NKG2D on T cells infiltrating the intestine, where they can target the epithelial causing damage and possibly the release of self-antigens activating T cells and amplifying the bystander T-cell response (57). IL-15 can also play an important role in the migration of bystander memory CD8 T cells to the site of inflammation. During acute hepatitis A, IL-15 upregulates CCR5 chemokine receptor enhancing their requirement for liver parenchyma (58) (**Figure 3A**). The synergy between cytokines plays an important role in bystander T-cell activation. For instance, both IL-23 and IL-1β can act synergistically to induce the expression of IFNγ, IL-22, and GM-CSF in effector/memory CD4 T cells (59–61). Another example of cytokine synergy and bystander activation is depicted in IL-2 and IL-33. Indeed, IL-2 upregulates IL-33R (ST2) in Th2 CD4 T cells making them express IL-13 (61). Furthermore, the IL-1 family member proinflammatory cytokine IL-18 can induce the expression of IFNγ from naive and memory CD4 T cells and IL-17 from Th17 CD4 T cells in an antigen-independent manner (59, 61–63). In response to IL-18, IL-18Rα^+^ memory CD4 T cells express a wide spectrum of cytokines including IL-5, IL-6, IL-13, IL-22, GM-CSF, TNFα, and IFNγ (64). The upregulation of IL-18Rα can be attributed to IL-12, which acts synergistically with IL-18 and induces Th1 and Th17 to express IFNγ in a TCR-independent manner (59, 61). Finally, high doses of IL-2 override the requirement of TCR stimulation and synthesize naive CD4 T cells to become responsive to IL-12 and IL-18 and acquire a Th1 phenotype and express IFNγ (62).

Lymphopenic conditions following chemo- and radiotherapy represent another source of cytokines, where these conditions result in a significant decrease in the pool of T cells. Consequently, there is an increase in the bioavailability of homeostatic cytokines including IL-7 and IL-15, which results in the expansion of T cells, a process called lymphopenia-induced homeostatic proliferation (LIP) (17, 65–69). Regarding naive CD8 T cells, they can actually proliferate and acquire a memory-like phenotype and functions in response to lymphopenia in a self-peptide/MHC-dependent manner, independent of non-self antigens (**Figure 2A**) (15, 16, 44, 45, 70–74). Under steady-state conditions, naive CD8 T cells can also acquire a memory-like phenotype in the presence of IL-15 and the transcription factor EOMES (75–77). These antigen-inexperienced cells are termed “virtual memory” T cells (T_VM_) (10, 75, 77, 78), which accumulate during aging (79–81). Later, it was demonstrated that naive T cells that undergo LIP are phenotypically very similar to T_VM_ (77, 82). In summary, these studies demonstrated the importance of cytokines along with tonic TCR stimulation in the activation of naive CD8 T cells. Hence, we speculate that naive T cells can also undergo another type of bystander activation that is different from memory CD8 T cells, i.e., dependent on both cytokines and the self-peptide/MHC complex.

One can speculate that this type of differential requirement could be attributed to the epigenetic programming specific to each cell population making memory T cells poised for rapid recall of effector responses compared to naive CD8 T cells. In fact, human memory CD8 T cells maintain permissively an open unmethylated state of promoter regions associated with effector molecules and cytokines (e.g., IFNγ and Prf1) during homeostatic proliferation compared to naive CD8 T cells (83, 84). Furthermore, naive CD8 T cells were not able to express IFNγ following classical *in-vitro* TCR/CD28 stimulation even in the presence of common gamma-chain cytokines (85). However, they upregulate the activation marker CD38 and express IFNγ in the presence of anti-CD3/CD28 antibodies and the proinflammatory cytokine IL-12 (85).

#### 2.1.2 The role of the peptide/MHC complex in naive and memory T-cell activation

As we have discussed earlier, naive CD8 T cells can proliferate and upregulate T-cell activation markers under lymphopenic conditions (**Figure 2A**), which suggests the importance of cytokines in LIP (86). However, they still require an interaction with the self-peptide MHC complex, where adoptive transfer of FACS-purified P14 naive CD8 T cells (LCMV antigen-specific CD8 T cells) into irradiated MHC-I-deficient mice failed to induce such proliferation and activation compared to WT mice (15). Furthermore, Cho et al. demonstrated that naive CD8 T cells failed to undergo proliferation upon transfer into irradiated MHC-I KO and TAP-1 KO mice compared to WT mice (13). These findings suggest the requirement for a self-peptide/MHC-I complex to drive such proliferation and activation of naive T cells, hinting toward a link between the self-peptide/MHC complex and responsiveness to cytokines. Indeed, Stoklasek et al. demonstrated that adoptive transfer of naive OT-1 CD8 T cells into irradiated MHC-I-deficient mice failed to undergo proliferation even after IL-15/IL15Rα treatment, suggesting the need for MHC-I interaction for an adequate IL-15 response in naive CD8 T cells (87). Along the same lines, LCMV acute infection results in the upregulation of CD122 (IL-2/IL-15Rβ) in CD8 T cells (88). Thus far, the abovementioned observations give us a clue toward the role of the self-peptide/MHC–TCR axis possibly by sensitizing naive cells to be responsive to common gamma chain cytokines. Although it is clear that cytokines play an important role in memory T-cell bystander activation, Goplen et al. demonstrated the activation of the downstream TCR signaling following bystander activation of memory T cells (14). In fact, the proinflammatory cytokines, such as IL-12, TNFα, and IL-15 that typically drive bystander memory T-cell activation, lead to the phosphorylation of CD3ε chain that propagated downstream the signaling pathway of TCR including ZAP70 and its downstream messengers LAT. Hence, this study suggested that proinflammatory cytokines make use of TCR/CD3 signalosome to facilitate bystander effector/memory CD8 T-cell responses. To elucidate the role of MHC in the bystander activation of memory CD8 T cells, the authors adoptively transfer VSV-specific CD8 memory T cells to congenic MHC class I-sufficient or -deficient hosts followed by infection with unrelated infection (*Listeria monocytogenes*—Lm) showing a reduction in IFNγ expression in MHC-I-deficient mice compared to WT mice (14). These data suggest the importance of MHC-I in the bystander activation of memory CD8 T cells. The obvious question is “Can these cells protect against unrelated infection?” A direct experiment will be the adoptive transfer of antigen-specific memory T cells to naive mice followed by infection with an unrelated pathogen. For instance, Soudja et al. showed that the adoptive transfer of LCMV polyclonal memory CD8 T cells or OT-1 memory CD8 T cells from mice infected with Lm-Ova decreases the bacterial load of Lm in WT mice (89). Similarly, Berg et al. observed protection against Lm using vaccinia virus expressing Ova (VacV-Ova) as a primary infection (23).

## 3 Biological significance of bystander T cell activation

While bystander T-cell activation is widely described in humans and multiple mouse models (10, 12), the function of bystander T cells could vary from one disease to another, i.e., protective (23, 43, 89) or pathogenic (90–94). Furthermore, other studies did not observe a significant contribution of the process toward pathogen clearance (35, 95, 96). This might be attributed to the masking effect of other cell types including γδ T cells, ILCs, and unconventional memory T cells contributing to the ongoing immune response (10). The lack of a consensus agreement on the function of bystander T cells could result from the usage of different preclinical experimental mouse infection models. In the next sections, we will discuss the role of bystander T cell activation not only during immune responses against pathogens but also in cancer, atherosclerosis, autoimmunity, and transplantation.

### 3.1 Bystander T cell activation during microbial infections

The occurrence of bystander T-cell activation has been well-documented in human infectious diseases (4). For instance, in patients with primary human immunodeficiency virus (HIV) and hepatitis B virus (HBV) infections, a robust T-cell activation was detected not only in antigen-specific CD8 T cells but also in EBV, CMV, and Flu-specific CD8 T cells (97, 98). Furthermore, HIV patients with interrupted antiretroviral treatment are characterized by activation of antigen-specific and non-specific CTLs (99).

These observations have been attributed to the common gamma chain cytokine IL-15 in the expansion and activation of bystander T cells (98, 100). Yet, the remaining question still remain “What role do these bystander T cells play during infection?” Is it protective or pathogenic? In the case of HBV, bystander-activated CD8 T cells might not contribute to HBV clearance but are implicated in precipitating the immunopathology associated with viral hepatitis. Those cells contribute to the hepatic parenchymal injury, characteristic of chronic viral hepatitis (101). Along the same lines, acute hepatitis A viral (HAV) infection is associated with severe hepatic parenchymal injury that was thought to be precipitated by HAV-specific CD8 T cells (**Figure 3A**) (102). However, in chimpanzees, HAV-specific CD8 T cells fail to produce significant levels of IFNγ or CD107a post-stimulation with HAV-specific peptides, demonstrating the hypofunction of viral-specific CTLs (103). To further understand the role of CD8 T cells during HAV infection, Kim et al. (55) assessed the specificity of activated CD8 T cells during acute HAV infection. They found that a substantial proportion of activated CD38^+^ HLA-DR^+^ CD8 T cells in the periphery of acutely HAV-infected patients were unrelated to HAV but specific against Flu, EBV, CMV, vaccinia virus, and respiratory syncytial virus (55), echoing earlier observations in HIV and HBV patients. Additionally, activation of bystander CMV- and EBV-specific CD8 T cells has been associated with acute pediatric hepatitis E virus (HEV) (104). Furthermore, there was an increase in the frequency of polyclonal Th1 CD4 T cells and CD8 T cells in the peripheral blood (104).

These observations pose the question “What is the mechanism behind the expansion and activation of non-cognate antigen-specific “bystander” T cells? Increasing levels of IL-15 and IL-18 have been observed in the sera of acutely HAV- and HEV-infected patients, suggesting a role of these cytokines in the antigen-independent activation of unrelated virus-specific CD8 T cells in these patients (55, 104). Furthermore, infecting human hepatoma cell lines (HepG2) with HAV resulted in the production of high levels of IL-15, which has been shown to drive HAV-unrelated CD8 T-cell activation (upregulation of NKG2D) in the absence of cognate antigenic stimulation (55). To evaluate the role of these bystander activated CD8 T cells in inducing liver tissue damage, the authors showed that IL-15-activated CD8 T cells isolated from healthy controls and intrahepatic CD8 T cells isolated from the livers of acute hepatitis A (AHA) patients were capable of lysing K562 target cells which do not express MHC class I and also lysing liver-derived Huh-7 cells (55). Furthermore, CMV-specific CD8 T cells isolated from the periphery of AHA patients but not healthy adults were able to lyse K562 cells (55). These results suggested that the hepatic injury associated with AHA infection is TCR independent and can be induced by IL-15-activated HAV-unrelated CD8 T cells in an NKG2D-dependent mechanism. Indeed, the group was able to infer a positive correlation between the proportion of activated HAV-unrelated virus-specific CD8 T cells and liver tissue damage during acute hepatitis A infection.

Another example showing the contribution of bystander T-cell activation to viral infection is the zoonotic viral hemorrhagic fever caused by the Lassa virus which is endemic to western Africa. Using a bone marrow chimera mouse model followed by Lassa viral infection, the authors demonstrated the activation of polyclonal CD8 T cells as well as OT-1 cells which is specific to ovalbumin (105). Along the same lines, in asymptomatic or mild symptomatic coronavirus disease 2019 (COVID-19) patients, there is a burst of COVID-19 non-specific CD8 T cells compared to severe symptomatic patients (**Figure 3B**). This early expansion of bystander CD8 T cells could play a role in the prevention of disease progression. Harnessing this non-specific response early during COVID-19 could be a better strategy to avoid disease progression (106). This work provided an up-close mechanistic insight on the role of bystander-activated T cells in the immunopathology of a disease.

### 3.2 Role of bystander T cells in cancer

It is widely accepted that tumor-infiltrating T cells (TILs) are heterogeneous in their specificity, i.e., a hybrid of both tumor-specific and non-specific T cells, where the proportion of tumor antigen-specific CD8 T cells (CTLs) among the TILs varies depending on the tumor type (107–110). For instance, melanoma tumors might harbor 50%–80% tumor-reactive CTLs out of their TILs. On the other hand, TILs of cold tumors such as serous ovarian cancer and microsatellite-stable colorectal cancers lack a tumor-reactive T-cell repertoire (107, 109, 110). The TILs of such tumors harbor some viral-specific T cells such as EBV-reactive CD8 T cells (110). Similarly, glioblastoma, renal cell carcinoma, endometrial carcinoma, head and neck, thyroid, and breast cancers house EBV, CMV, and Flu-specific memory CD8 T cells (108).

#### 3.2.1 Bystander T cell recruitment

Recruitment of these bystander CD8 T cells to the tumor microenvironment is mostly driven by chemokines and inflammatory cues enriched in the tumoral niche and independent of a cognate antigen (111, 112). The chemokines can be secreted from tumor cells such as CCL5 secreted by the cancer cells. Next, CXCL9 produced from IFNγ-stimulated antigen-presenting cells recruits T cells expressing CXCR3. CXCL10 is also involved in the process of recruiting CXCR3^+^ T cells, which are mostly bystander in nature (113). However, tumor-specific T cells can also express both chemokine receptors (CXCR3 and CCR5), which suggests that the recruitment of bystander T cells is simply a collateral phenomenon accompanied by recruitment of tumor-specific T cells (114). In both scenarios, bystander T cells infiltrate and accumulate in the tumor (111, 112).

#### 3.2.2 Approaches to harness bystander T cells

The detection of non-exhausted viral-specific T cells in various tumors and their correlation with better tumor control (115, 116) encouraged the idea of harnessing their cytotoxicity especially that a lot of tumoral antigens are either self or modified self-antigens that fail to mount a strong TCR stimulation (117–119).

##### 3.2.2.1 Vaccination and infection mouse models

One way to fulfill this aim is by establishing OT-1 chimeras through infecting wild-type naive mice that harbor congenic OT-1 CD8 T cells with VSV-expressing OVA. This approach results in mounting OT-1 memory CD8 T-cell response 30+ days post-infection (108). Injecting VSV viral peptide reactivated the OT-1 memory T cells, which is associated with delayed B16 melanoma growth. Furthermore, treating tumor-bearing mice with systemic anti-PDL-1 antibodies along with VSV-OVA peptide intratumorally resulted in the eradication of B16 tumors in 34% of the mice (108). These data suggested that activation of bystander memory viral-specific T cells enhances antitumor immunity. This effect is even enhanced with PD-L1 blockade. Indeed, using PD-1KO mouse models or blocking the PD-1/PD-L1 axis enhances the functionality of bystander T cells but reduces the longevity of T cells (120). Along the same lines, intratumoral treatment of melanoma with heat-inactivated Flu virus resulted in reduced tumor growth and increased CD8 T-cell infiltration. Such treatment was shown to sensitize “cold” non-immunogenic tumors to checkpoint immunotherapy, rendering them “hot” and responsive tumors (121). Even at a transcriptomic level, Caushi et al. showed the enrichment of virus-specific CD8 T cells in tumor tissues of lung cancer patients who received immune checkpoint blockade. Those viral-specific CD8 T cells expressed effector genes higher than tumor-specific CD8 T cells (122). These results suggested that the concurrence of infection and cancer can lead to the activation of pre-existing viral-specific CD8 T cells in the tumor microenvironment in a TCR-independent manner.

##### 3.2.2.2 Cytokines and chimeric antigen receptor T cell-based approaches

Another way to activate bystander T cells is through cytokine-based immunotherapy and chimeric antigen receptor (CAR) T cells. Indeed, injecting mice-bearing tumors (B16, 3LL, or Renca) with anti-CD40/IL-2 resulted in vigorous expansion of memory CD44^hi^ NKG2D^+^ CD8 T cells and significant protective anti-tumoral effect (123). Recently, it has been demonstrated that CTLs can kill tumors that downregulate MHC-I through the NKG2D–NKG2DL axis (124). Furthermore, the IL-15-based superagonist (ALT-803) results in an antigen-independent enrichment of innate-like CD8 T cells expressing NKG2D with an antitumor activity (125). *In vivo*, ALT-803 can eliminate myelomas from the bone marrow providing survival benefit for the treated mice (125). These studies suggest that the anti-tumoral effect can be induced by cytokine-based immunotherapy and is mediated by the expanded NKG2D^+^ memory CD8 T cells in an MHC-independent manner. Additionally, CAR T cells can be designed in a way specific to both CD3 chain and ubiquitous tumor antigen such as EphA2 (126), which is commonly expressed by glioblastoma, lung, breast, and prostate cancers (127, 128). Hence, these “engager” cells are not only directed toward a tumor but also can activate bystander T cells (126). Along the same lines, using the scRNA-Seq approach, Kaminiski demonstrated for the first time the transcriptome signature of CARneg CD8 bystander T cells following CAR therapy for B-cell leukemia enrich for NK-like markers (CD160, KLRD1, and KIR3DL2) and chemokines and chemokine receptors (CCR9 and CCL5) and less of naive signature (129).

The obvious question here is “Is there any role for bystander CD4 T cells?” Can CD4 and CD8 cooperate to enhance tumor killing? Lee et al. discussed nicely the role of bystander CD4 T cells in infection, autoimmunity, and cancer (130). Furthermore, Joncker et al. co-injected TCR transgenic CD4 and CD8 T cells that can recognize HY male antigen into female mice bearing fibrosarcoma tumors. Following priming of the mice with male cells, there were accumulation and expansion of both Tg CD4 and CD8 T cells, which were considered bystander, into the tumor and tumor-draining lymph nodes (131). Furthermore, Schietinger et al. showed the cooperation between CD4 and CD8 T cells as a requirement for bystander killing of tumor cells using transgenic mouse models (132). Taken together, the aforementioned studies suggest an active contribution of the bystander CD4 and CD8 T cells among TILs to the anti-tumor immunity in mice and humans.

### 3.3 Bystander T cells and auto- and alloimmunity

Auto- and alloimmune responses are considered two faces for the same coin. In autoimmune disorders, immune responses are dysregulated resulting in the persistence and activation of self-reactive T cells causing disease sequalae (133). However, the initial events and propagation of autoimmunity are still not very well understood. In the case of alloimmunity, graft rejection results from the recognition of non-self antigens associated with the donor organ (134). Hence, both immune responses resulted from the break in self-tolerance, but the cause behind them is different. One is through missing self and the development of autoimmunity, while in the case of graft rejection, it is the recognition of non-self antigens within the donor graft.

#### 3.3.1 Bystander T cells in autoimmune diseases

During a vigorous immune response mediated by an infection, vaccination, or even alloantigens, bystander activation of low-affinity self-reactive CD8 T cells that escaped thymic negative selection seems to be a plausible hypothesis for autoimmunity that has been assessed and studied for the past few decades. For instance, this can be explained by the onset or recurrence of autoimmune disorders following vaccination or solid organ transplantation (135–140). The contribution of bystander activation to autoimmune disease pathogenesis stems from early seminal work on a herpetic stromal keratitis (HSK) preclinical mouse model, an autoimmune disorder resulting from corneal infection with HSV-1. HSV-1-infected OVA transgenic RAG^−/−^ or SCID mice whose TCR is reactive against the OVA peptide only resulted in HSK. These results suggested that autoimmunity does not require a TCR-mediated activation and demonstrated that this activation of T cells is beyond antigen specificity (141, 142).

##### 3.3.1.1 Multiple sclerosis

Multiple sclerosis (MS) is essentially caused by autoimmune-mediated demyelination of neurons of the central nervous system. Disease relapse or exacerbation has been associated with recent microbial infections (143). Although thought to be mainly driven by activated myelin protein self-reactive T cells such as myelin oligodendritic glycoprotein (MOG)-specific T cells that are cross-reactive to microbial epitopes, more recent studies evaluating the pathogenesis of the disease in murine model known as experimental autoimmune encephalitis (EAE) demonstrated that bystander activation of autoreactive T cells stands as an alternative mechanism for MS pathogenesis. For instance, majority of the CD4 T cells infiltrating the central nervous system (CNS) are not specific to MOG (60), where they are mainly expressing cytokines related to Th17 cells including IL-17A, IFNγ, and GM-CSF (**Figure 4A**). Suppression of these cells can be mediated by Tregs. Indeed, Kim et al. showed that engineered myelin basic protein (MBP)-specific Tregs had the capacity to suppress not only antigen-specific T cells but also non-specific ones (144). Furthermore, other studies showed that Tregs can suppress T cells in an antigen-independent manner (145, 146). The obvious question is “How can these cells get activated in a TCR-independent fashion?” Indeed, the IL-1 family member IL-1β induces CD4 T cells to express GM-CSF and the transcription factor Bhlh40. Furthermore, human CD4 T cells isolated from an MS patient expressed IL-1R1 as well as TLR2 and TLR4, which contributed also to the production of IL-6, IL-17A, IFNγ, and GM-CSF (147). Nogai et al. showed that, in a CD4 T-cell model, treating transgenic mice that only recognize the MBP Ac1-11 peptide with LPS resulted in the development of EAE (148). Intriguingly, CD4 bystander T cells can also play a neuroprotective role as demonstrated by other labs (149). This kind of protection is mainly mediated by IL-4, suggesting that Th2-mediated immune response could be protective against EAE (149). Furthermore, the IL-12 family member IL-27 can induce the upregulation of PD-L1 in CD4 T cells ameliorating EAE (150). The damage of host cells as result of the ongoing immune response can result in the release of new self-antigens, a phenomenon called epitope (determinant) spreading. The recognition of self-antigens results in autoimmunity (151). For instance, in relapsing–remitting experimental autoimmune encephalomyelitis (R-EAE), a model for MS, epitope spreading plays a crucial role in disease exacerbation, where T cells are initially activated by specific immunodominant epitopes, such as PLP (139–151) or MBP (84–104). However, damage to the CNS exposes additional myelin epitopes that were not originally targeted (intermolecular epitope spreading). On the other hand, intramolecular epitope spreading occurs with the same protein. In this scenario, an autoantibody can bind to the same protein but at a different protein region compared to initial binding (152). Furthermore, there are wide varieties of pancreatic tissue antigen GAD-65 and proinsulin in type 1 diabetes patients compared to healthy controls supporting intramolecular epitope spreading (153).

##### 3.3.1.2 Type 1 diabetes

Type 1 diabetes (T1D) is characterized by self-reactive T cells that damage insulin-secreting beta cells in the pancreatic islet of Langerhans (154). Although the initial trigger of the disease is not completely understood, viral infections mediated by the Coxsackie virus as well as other viruses (**Figure 4B**) can contribute to beta-cell destruction via a) direct infection of beta cells, b) inflammatory cytokines, and c) molecular mimicry where viral epitopes overlap with autoantigens (155–158). Majority of the T cells in the islets are “bystanders” non-specific to islet antigens and mainly play a protective role by reducing the accessibility of the antigen-specific T cells to the autoantigens (159). Furthermore, several studies demonstrated the pathogenic role of innate-like T cells including MAIT, γδ T cells, and iNKT cells in T1D (160–162) (**Figure 4B**).

##### 3.3.1.3 Rheumatoid arthritis

Bystander activation of CD8 T cells has been proposed in a wide spectrum of autoimmune diseases including rheumatoid arthritis (RA) and celiac disease (57, 163–165). Indeed, EBV- and CMV-specific activated CD8 T cells have been detected in the synovial fluid of rheumatoid arthritis patients (166) (**Figure 4C**). The activation of these unrelated CD8 T cells is thought to be mediated by a chronic inflammatory environment enriched in the synovial milieu. Indeed, TLR2 activation initiates RA through T cell IFNγ secretion in the absence of a specific antigen (167). Additionally, bacterial lipopolysaccharides can activate osteoclast contributing to the proinflammatory environment (168). Although it has been demonstrated that CD8 T cells expressing TLR4 correlates with disease severity and expression of effector cytokines/molecules (169), other reports suggested their regulatory function (170, 171).

In regard to CD4 T cells, NKG2D^+^ CD4 T cells are enriched in the peripheral blood and synovium of RA patients (163). The NKG2D ligand MICA/B is upregulated in the synovium in response to proinflammatory cytokines including TNFα, which hints a role of the NKG2D/NKG2DL axis in joint damage (163). RA can develop in another preclinical autoimmune disease model (Sjogren syndrome), suggesting a mechanism of bystander T cell activation via cross-reactivity; however, the authors did not rule out this possibility (172).

##### 3.3.1.4 Atherosclerosis

Over the last few years, the importance of autoimmunity has been widely accepted in the pathogenesis of atherosclerosis, a common pathology underlying cardiovascular diseases (CVDs) characterized by arterial wall plaque formation rich in lipids and immune cells (173). Indeed, atherosclerosis patients harbor circulating autoantibodies against apolipoprotein B containing lipoproteins (ApoB) such as LDL, which indicates loss of self-tolerance (174, 175). This actually served as an impetus for several labs to shift their efforts to understand the mechanisms underlying autoimmunity during atherosclerosis. For instance, two studies by Wang et al. and Depuydt et al. used a combination of scRNA-Seq and TCR-Seq to demonstrate the breakdown of peripheral tolerance in atherosclerosis (176, 177). In the mouse study of Wang et al., the highest degree of plaque-specific T-cell expansion was observed in the cytotoxic CD8 T-cell compartment expressing CD69 and Fosb, both indicating TCR signaling. Similar observations were documented in human subjects with coronary artery disease (CAD) (178). Additionally, the majority of CD8 T-cell clones within aortic plaques shared TCR patterns with those seen in aorta draining lymph nodes (RLNs) of an Apoe^−/−^ mouse atherosclerosis model (176). Other studies revealed the presence of ApoB-specific CD4 T cells in mice and patients with atherosclerosis (**Figure 4D**).

Although the abovementioned studies strongly suggest the autoimmune nature of atherosclerosis, the trigger of autoimmunity is not completely understood. However, few recent observations in the literature might explain the phenomenon. For instance, episodes of infections are correlated with CAD complications (179, 180). This process might involve CD8 cells found in atherosclerotic plaques (176, 177, 181–188). A small percentage of these cells have shown cross-reactivity to influenza virus, Epstein–Barr virus (EBV), and cytomegalovirus (CMV) in the plaque (189). Along the same lines, de Jong et al. found that ~2% of TCR sequences in the plaques were specific to viral epitopes, where the T cells exhibit signs of activation (190). To investigate if this process happens through molecular mimicry, the authors eluted peptides from MHC-I molecules followed by mass spectrometry. They observed several peptides that could be cross-reactive; however, it has not been tested if these peptides can stimulate T cells from the plaque. An alternative explanation could be the activation of the plaque T cells in an antigen-independent manner, where cytokines secreted during inflammation can activate T cells in the plaque non-specifically.

##### 3.3.1.5 Systemic lupus erythematosus

Systemic lupus erythematosus (SLE) is a complex autoimmune disease characterized by a defect in the clearance of apoptotic cells, which results in the breakdown of self-tolerance and the production of autoantibodies against dsDNA leading to system organ damage including the kidneys (191, 192). Activated bystander CD4 T cells expressing NKG2D secrete IL-10 and TGF-β, suggesting an immunoregulatory role in SLE (193). Furthermore, *in vitro*, these cells can inhibit T cell proliferation (193). On the contrary, *Dermatophagoides pteronyssinus* group 2 (Der p2), a main allergen for house dust mite, causes the upregulation of proinflammatory cytokines from PBMCs of allergic SLE patients. This stimulation can activate B cells for autoantibody production in a bystander manner (194).

#### 3.3.2 Bystander T cells in transplantation

In transplantation, the role of bystander T-cell activation has not been widely discussed; however, several studies reported the significant effect of TLR agonists and microbial infections on graft rejection in preclinical mouse models (195–200). For instance, Welsh et al. showed that despite treatment of thymectomized mice with the tolerance induction regimen (co-stimulation blockade, e.g., anti-CD154 and donor-specific leukocyte), which classically induces skin graft survival for more than 100 days, acute LCMV infection precipitates graft rejection (200). Furthermore, TLR agonist treatment results in rapid skin allograft rejection despite the co-stimulation blockade treatment (197, 198). In a human cardiac allograft vasculopathy (CAV) study, the authors suggest the infiltration of bystander T cells into the graft (201).

### 3.4 Therapeutic implications of bystander T-cell activation

As we have discussed earlier in the previous sections, bystander T cell activation can lead to a pathological versus protective effect depending on the disease context. For instance, acute viral hepatitis infection is associated with liver parenchymal cell damage mainly mediated by bystander T cells (**Figure 3A**). Conversely, an early burst of bystander T-cell activation is associated with asymptomatic/mild COVID-19 symptoms but absent in patients with severe symptoms (**Figure 3B**). Hence, it seems plausible to first define the role of bystander T cells in a specific disease setting and then decide to harness or inhibit these cells. Although both disease settings are mediated by viral infections, it is not clear why bystander cells are protective in one scenario versus the other. One can speculate that the inflammatory environment and tissue type discrepancy could play a role.

**Figure 5:**
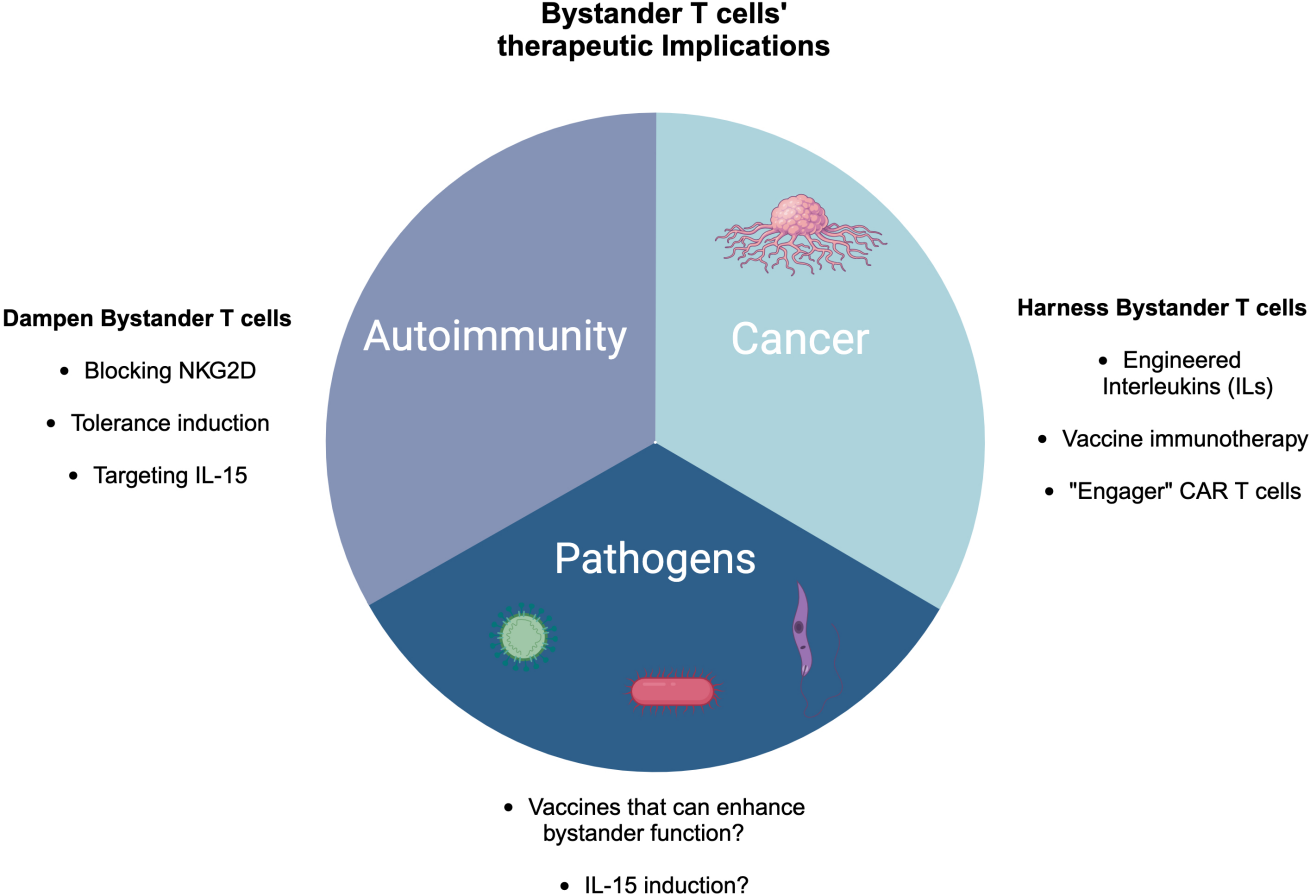
Therapeutic implications of bystander T cells in cancer, pathogen control, and autoimmunity. In cancer, there are several ways to harness bystander T cells including engineered cytokines, vaccine immunotherapy, and engager CAR T cells. In the case of autoimmunity, blocking NKG2D, targeting IL-15, and tolerance induction might be potential approaches to dampen bystander T cells, while in pathogen control, IL-15 induction and developing vaccines could be novel strategies.

Since bystander T cells are activated in an antigen-independent manner and lack antigen specificity, it is difficult to target them. However, the NK receptor NKG2D could be one of the surface proteins to target. Indeed, blocking NKG2D in HBV preclinical mouse model reduces liver injury (**Figure 5**) (202). On the contrary, during *Listeria monocytogenes* infection, blocking NKG2D exacerbates the disease, suggesting a protective role of NKG2D^+^ cells specifically CD8 T cells (11, 43). In the case of COVID-19 patients, the phenomenon of early bystander burst T-cell activation can be exploited to avoid disease progression and induce early viral control (106). However, it is not clear why a cohort of patients develop this early bystander activation while others do not.

The heterogeneity of TILs along with the expression of NKG2D can be exploited in various ways to harness anti-tumor immunity. For instance, cytokine-induced killer cell therapy can be one approach where a cocktail of cytokines including IL-2 and IFNγ can be used to upregulate NK cell receptors such as NKG2D (203, 204). Similarly, engineered interleukins (ILs) such as IL-15 and a combination of IL-2 and anti-CD40 can enhance expansion and induce cancer cell killing via NKG2D in a TCR-independent manner (114, 123, 125, 205, 206) (**Figure 5**).

Since pathogen-derived peptides are widely expressed by tumors, such as glioblastoma, expressing CMV antigens, while melanoma is rich in bacterial antigens, harnessing viral-specific bystander T cells within the tumor microenvironment via vaccination-based immunotherapies could be an alternative strategy (**Figure 5**). For example, active influenza vaccine enhanced lung cancer outcomes in both mouse models and human patients (121). Additionally, the study revealed that intratumoral vaccination with heat-inactivated influenza virus notably reduced skin melanoma and improved survival rates (105). CAR T cells with dual TCR (one for the tumor antigen and virus-specific receptor) could even be a better strategy to enhance the efficacy of tumor killing (114, 207) (**Figure 5**).

### 3.5 Future directions and concluding remarks

Antigen specificity is the hallmark of the adaptive immune response (208); but, view less the existence of innate memory has been recently described during alloimmune responses (209, 210). However, antigen non-specific T cells can proliferate, get activated, and acquire effector functions in an inflammatory milieu. The latter is referred to as bystander activation. Bystander activation has been always been seen as an alternative TCR-independent activation pathway of memory T cells. Regarding naive T cells, they are responsive to cytokines and require a self-peptide–MHC engagement to induce their activation and proliferation. This phenomenon called homeostatic proliferation, which occurs in lymphopenic conditions such as irradiation-induced bone marrow ablation, HIV infection, and other lymphopenia-inducing processes, could be the naive T-cell counterpart version of bystander activation. The differential requirement of cytokines versus both tonic TCR signaling and cytokines could be attributed to epigenetic programs acquired during the development of both T-cell subsets (naive vs. memory). Although the mechanisms underlying bystander T-cell activation are well documented, the function can vary depending on the human disease setting or the preclinical animal model as we have discussed earlier in several examples such as microbial infection, cancer, autoimmunity, and transplantation.

To exploit the function of bystander T cells in the context of autoimmunity, we can draw parallels from tumor immunity and microbial infections. However, the disease setting is different. For instance, in cancer, several studies as discussed above seek approaches to harness the effector functions of bystander T cells in the tumor microenvironment (**Figure 5**). Indeed, bystander T cells do not exhibit an exhaustion signature compared to tumor-specific T cells in melanoma patients (116). However, in autoimmunity, strategies should be developed to dampen the cytotoxicity of autoreactive T cells or induce tolerance. Similar to microbial infections, anti-NKG2D could be one approach to inhibit bystander T cells in the context of autoimmunity (**Figure 5**). However, the approach of one-size-fits-all cannot be applied to autoimmunity since some autoimmune diseases are characterized by immunoregulatory bystander T cells such as in the case of SLE where activated bystander CD4 T cells expressing NKG2D secrete IL-10 and TGF-β (193). Furthermore, bystander CD4 T cells in MS can play a neuroprotective role as well (149). Taken together, utilizing bystander T cells in cell therapy represents a potential opportunity as a future perspective where harnessing tumor immunity is a great example. However, in the case of other diseases such as autoimmunity, the approach should be tailored based on the disease context, the tissue environment, and other factors. Finally, bystander T cells could play an important role in designing novel vaccines, enhancing their efficacy.
